# 

**Published:** 1889-06

**Authors:** 


### TABLE OF CONTENTS.

ORIGINAL AND SELECTED.

Antipyrin ........ 201
Parecentesis in Internal Hydrocephalus ........ 207
Artificial Fecundation, &c. ........ 209
A case of Laparotomy, &c. ........ 213

ABSTRACTS &c.

Chlorate of Potash in Epithelioma; Accidents Incidental to the use of the exploring needle .. 215
The Hypod. use of Ergot in Facial Neuralgia ... 216
Medicinal treatment of Intestinal obstruction .. 217
Crude Petroleum as a surgical dressing; Action of iodide and brom. potas. upon morphine .. 218
Microbe as a factor in disease; Prognosis of locomotor ataxia; Phenacetine ........ 219
Chloroform in Dyspepsia ........ 220
Cancer treated by creosote; Treat. of typhlitic diseases ........ 221
Headache; Local treat. diphtheria by antipyrine; Hydrate of chloral in chapped nipple .. 222
Sweet spirits of nitre a solvent of salicylic acid; Exalgine, the new analgesic ........ 223
The disinfection of surgical instruments ........ 224
Reflex neuralgia caused by tape worm; Treatment of insomnia ........ 225
A new remedy for cholera ........ 226
Medicinal treatment of menstrual disorders ... 227
Carbolic acid inhalations in whooping cough; Permanganate of potas. in rattlesnake bites; Bloodless treat. of ingrowing nail; M. Pasteur 228
Useful formula in skin disease; For rigid os and perineum; To remove hiccough; Chamomilla 259
Prostatic enlargement; What prohibition does; Acetanilide an external sedative; Gelsemium 230

SCIENTIFIC.

The Anæsthetic revelation ........ 231
Sixty miles to the Moon; Wonderful photographic feat; Microscopic examination, &c. ..... 232

NOTES AND FORMULÆ.

For dysentery in children; Dysentery in the adult; To abort bronchitis; Gargle to prevent loosening the teeth ........ 233
Cough mixture; Dysmenorrhœa; For eczema of the anus and genitals; Itch; Ulceration of the ear; For dysentery of children; For dysentery of teething children ........ 234

EDITORIAL.

Negro dentists; The orator-A mistake; Lactucarium syrup and paste ........ 235
Lessons in nursing; Electricity, &c.; Handbook of Phthiscology ........ 236
Kirk's hand-book of physiology; Extra uterine pregnancy ........ 237
Psychic life of micro-organisms; May Homiletic Review; Cocaine; Laparotomy for peritonitis; Koller of Austria ........ 238
Special Notes ........ 239-240

